# Predictive utility of the multi-theory model in physical activity initiation and maintenance intentions among maintenance hemodialysis patients: a cross-sectional study

**DOI:** 10.3389/fpsyg.2026.1752207

**Published:** 2026-03-06

**Authors:** Rui Zhang, Yuchen Chu, Tianxin Liu, Peiying Wang, Yunfeng Li, Jinrong Gai

**Affiliations:** 1 School of Nursing, Shandong Second Medical University, Weifang, China; 2 School of Nursing, Shandong First Medical University, Jinan, China; 3 Department of Quality Control, The First Affiliated Hospital of Shandong First Medical University, Jinan, China; 4 Department of Scientific Research, The First Affiliated Hospital of Shandong First Medical University, Jinan, China

**Keywords:** initiation, intention, maintenance, maintenance hemodialysis, multi-theory model, physical activity

## Abstract

**Background:**

Physical activity (PA) reduces cardiovascular and all-cause mortality in patients undergoing maintenance hemodialysis (MHD); however, PA participation among this population remains suboptimal. As a fourth-generation health behavior theory, the Multi-Theory Model (MTM) uniquely defines independent determinants of behavior initiation and maintenance. However, its applicability in predicting PA behaviors among MHD patients remains unexplored. This study aims to evaluate the predictive validity of MTM constructs for initiating and maintaining PA intentions among MHD patients, providing evidence for developing targeted intervention strategies.

**Methods:**

A cross-sectional study was conducted from December 2024 to March 2025 at the hemodialysis center of a tertiary hospital in Shandong Province, China. A convenience sample resulted in 274 valid responses. A self-developed questionnaire collected sociodemographic and disease-related data, while the Measuring Change in Physical Activity Questionnaire assessed six MTM constructs. Correlation analysis and hierarchical multiple linear regression explored predictive relationships between MTM constructs and intentions to initiate and maintain PA.

**Results:**

After controlling for demographic and disease-related covariates, dialogue advantages (*β* = 0.238, *p <* 0.001), dialogue disadvantages (*β* = −0.087, *p* = 0.006), behavioral confidence (*β* = 0.560, *p <* 0.001), and changes in physical environment (*β* = 0.180, *p <* 0.001) from the MTM initiation model significantly predicted PA initiation intention, explaining an additional 56.9% of variance (ΔR^2^ = 0.569, *p <* 0.001). Within the MTM maintenance model, emotional transformation (*β* = 0.390, *p <* 0.001), practice for change (*β* = 0.398, *p <* 0.001), and changes in social environment (*β* = 0.131, *p* = 0.004) significantly predicted PA maintenance intention, accounting for an additional 53.7% of variance (ΔR^2^ = 0.537, *p <* 0.001).

**Conclusion:**

This study demonstrates the utility of the MTM in predicting PA intentions among MHD patients, particularly in identifying factors related to the initiation and maintenance stages. The MTM framework offers a two-stage perspective on behavior change, contributing to the understanding of key factors associated with PA intentions. Future research could further explore the mechanisms underlying these factors in the broader population of MHD patients.

## 1 Introduction

End-stage renal disease (ESRD) is a severe chronic kidney disorder, with global prevalence steadily increasing. According to 2023 Global Burden of Disease data (Rafferty et al., 2025), approximately 4.59 million people worldwide are receiving kidney failure replacement therapy, with an age-standardized prevalence of 50.7 per 100,000. High-income regions show a prevalence of 111 per 100,000, while sub-Saharan Africa has the lowest at 3.8 per 100,000. For specific countries, the age-standardized prevalence is 176 per 100,000 in the USA, and 53.1 per 100,000 in China, with a total of approximately 1.18 million patients in China. With the aging population and rising prevalence of chronic diseases, the global disease burden of ESRD demonstrates an accelerating growth trajectory (Xie et al., 2025). MHD, as the primary renal replacement therapy for ESRD, significantly prolongs patient survival.

Despite continuous advances in dialysis technology and pharmacological treatments, the prognosis for MHD patients remains suboptimal. This population exhibits a substantially higher incidence of cardiovascular disease compared to the general population, with cardiovascular complications representing the leading cause of mortality (You et al., 2025). Furthermore, MHD patients commonly experience functional decline, chronic pain, and anxiety-depression disorders, which severely compromise their activities of daily living and social functioning (Dreiher et al., 2021; Li et al., 2023). Of particular concern is the widespread prevalence of sedentary behavior and low PA levels among MHD patients (Pengpid et al., 2023; Han et al., 2025), while insufficient PA demonstrably associated with significantly elevated all-cause mortality risk (Zhang et al., 2022).

Adequate PA offers multifaceted health benefits for MHD patients. Research indicates that PA is closely associated with improved patient prognosis and represents a promising approach to enhancing clinical outcomes (Zhao et al., 2025; Zhang et al., 2021). Moderate PA has been demonstrated to correlate significantly with reduced mortality in MHD patients (Liu et al., 2024), and higher levels of PA and regular exercise are positively associated with superior health-related quality of life (HRQOL) (Hu et al., 2024; Király et al., 2024). However, the current PA status among this population remains concerning. Most MHD patients exhibit low PA levels, primarily engaging in low-intensity activities (e.g., walking), and struggle to maintain regular exercise. Only a very limited proportion of patients achieve the recommended PA levels (≥150 min of moderate-intensity PA or ≥75 min of vigorous-intensity PA per week) (Hu et al., 2024). Consequently, assisting MHD patients in achieving and sustaining recommended PA levels should constitute an essential objective for improving their clinical outcomes and quality of life.

Exercise intention serves as a significant positive predictor of exercise behavior (Chen et al., 2022). Relevant research indicates that factors influencing PA intention among MHD patients primarily encompass several dimensions. First are physiological factors: MHD patients frequently present with multiple comorbidities (Wu et al., 2022a) and dialysis-related complications (Alshammari et al., 2025), imposing substantial physical burdens that may diminish PA motivation. Second are psychological factors: MHD patients’ perceptions of PA benefits versus barriers represent crucial determinants of participation willingness. When perceived benefits outweigh perceived barriers, individuals demonstrate greater inclination toward adopting health behaviors (Zhang et al., 2025). Additionally, external factors such as physical environment (Li et al., 2021) and social support (Zhang et al., 2025) exert significant influence on both the willingness and engagement of MHD patients in PA.

Currently, there remains a lack of systematic analysis regarding the multidimensional factors influencing the initiation and maintenance of PA behaviors among MHD patients. Given that this population is in long-term treatment, the key to PA lies not only in “starting” but also in the ability to sustain it. Previous health behavior theories, such as the Theory of Planned Behavior and Self-Determination Theory, have primarily focused on specific aspects of behavior change (Karloh et al., 2023). However, in chronic illness contexts, there has been relatively limited analysis of the emotional, self-regulation, and environmental support mechanisms needed for the maintenance phase, making it difficult to fully address the behavioral maintenance characteristics of MHD patients under the burden of ongoing treatment. As a fourth-generation health behavior theory, the MTM integrates key constructs from multiple classic theories and divides behavior change into two phases: initiation and maintenance, emphasizing that the determinants for each phase differ (Sharma et al., 2024). The initiation phase includes participatory dialogue, behavioral confidence, and changes in physical environment, while the maintenance phase includes emotional transformation, practice for change, and changes in social environment (Nahar et al., 2016). This distinction is crucial for MHD patients, as their PA initiation and long-term adherence may face different types of barriers and facilitators due to factors such as dialysis schedules, symptom fluctuations (e.g., fatigue), and psychosocial stress (Battaglia et al., 2024). MTM has demonstrated good explanatory and predictive value in PA and other health behaviors (Kapukotuwa et al., 2024), but its application for distinguishing and predicting PA initiation and maintenance in MHD patients remains limited. Therefore, this study aims to examine the predictive utility of MTM for PA initiation and maintenance intentions in MHD patients, identify phase-specific key determinants, and provide theoretical support for developing stage-based, targeted intervention strategies to address the different challenges this population faces in PA behavior change.

## 2 Materials and methods

### 2.1 Study design and population

This cross-sectional investigation used convenience sampling to enroll patients receiving hemodialysis at a tertiary hospital in Shandong Province, China, between December 2024 and March 2025. Eligible participants were adults (≥18 years) who had been on regular hemodialysis for at least 3 months, were ambulatory without assistance, were able to communicate and comprehend questionnaire items, reported < 150 min per week of moderate-intensity PA, and provided written informed consent. To minimize clinical heterogeneity attributable to vascular access–related complications and differences in care pathways, we included only patients whose hemodialysis vascular access was a native arteriovenous fistula (AVF). Individuals with a documented psychiatric disorder or Alzheimer’s disease, or with severe organic cardiac disease, cerebrovascular accident, or other major cardiovascular conditions were excluded.

### 2.2 Sample size

The sample size was determined using an *a priori* power analysis conducted with G*Power 3.1.9.7 software. To ensure sufficient statistical power for the most complex analysis in this study, calculations were based on the Initiation Intention Model, which included the maximum number of predictors (Total = 25: 21 dummy-coded covariates and 4 MTM constructs). The calculation used the F-test for linear multiple regression: fixed model, R^2^ deviation from zero, with the following parameters: a medium effect size (f^2^ = 0.15), an *α* error probability of 0.05, and a high statistical power (1 − *β*) of 0.95. The analysis indicated a minimum required sample size of 242 participants. To account for a potential invalid response or attrition rate of approximately 10%, the target sample size was adjusted to 267. A total of 280 paper questionnaires were distributed; 274 fully completed questionnaires were included in the analyses (response rate: 97.9%), and no missing data were present in the final dataset.

### 2.3 Study variables and instrument

#### 2.3.1 Demographic and clinical characteristics questionnaire

A self-designed questionnaire captured sociodemographic and clinical characteristics: age, gender, number of comorbidities, primary caregiver, monthly income, educational level, employment status, marital status, dialysis vintage, and dialysis frequency.

#### 2.3.2 Measuring change in physical activity questionnaire (MCPAQ)

This questionnaire was originally developed based on the MTM (Nahar et al., 2016) and subsequently adapted into a Chinese version by Yang et al. (2022). While the instrument has been validated for assessing PA intention in populations including hypertension patients (Yang et al., 2022) and pregnant women (Zhang et al., 2022), no previous studies have examined its predictive utility for PA intentions among MHD patients. The questionnaire comprises two subscales: the initiation subscale and the maintenance subscale.

The initiation subscale encompasses three dimensions: participatory dialogue (5 items assessing advantages and 5 items assessing disadvantages), behavioral confidence (5 items), and changes in physical environment (3 items). The participatory dialogue dimension score is calculated by subtracting the disadvantages score from the advantages score. For inferential analyses in this study, Dialogue Advantages and Dialogue Disadvantages were modeled as separate predictors (rather than a single difference score) to avoid the limitations of difference-score operationalization. The maintenance subscale includes three dimensions: emotional transformation (3 items), practice for change (3 items), and changes in social environment (3 items). All items are rated on a 5-point Likert scale ranging from “very uncertain” (0 points) to “very certain” (4 points), with higher scores indicating greater likelihood of initiating or maintaining PA.

Additionally, two single-item questions assess behavioral intentions: “How likely are you to engage in at least 150 min of moderate-intensity physical activity in the next week?” and “How likely are you to engage in at least 150 min of moderate-intensity physical activity every week going forward?” These items are designed to assess MHD patients’ intention or likelihood to initiate and maintain moderate-intensity PA. Importantly, the maintenance item captures a forward-looking intention to sustain guideline-level moderate-intensity PA over time, rather than maintenance of participants’ baseline PA level. The complete questionnaire consists of 29 items.

### 2.4 Data analysis

Data were analyzed using SPSS version 26.0. Descriptive statistics for categorical variables are presented as counts and percentages; continuous variables that satisfied normality are reported as mean ± standard deviation. Internal consistency of the questionnaire was evaluated using Cronbach’s alpha. Confirmatory factor analysis (CFA) was conducted based on the *a priori* MTM factor structure, with the initiation- and maintenance-phase measurement models tested separately. Model fit was evaluated using *χ*^2^/df, RMSEA, and fit indices including CFI, TLI, IFI, NFI, and GFI, using commonly cited criteria (e.g., *χ*^2^/df < 3, RMSEA < 0.08, and CFI/TLI/IFI/NFI/GFI > 0.90). Standardized factor loadings were inspected to evaluate item–factor relationships. Convergent validity was assessed using composite reliability (CR) and average variance extracted (AVE), and discriminant validity was evaluated using the Fornell–Larcker criterion. Associations between MTM constructs and intentions for PA initiation and maintenance were examined with Pearson correlation coefficients. To assess the contribution of MTM constructs to intention outcomes, hierarchical multiple linear regression models were constructed. Sensitivity analyses were conducted by collapsing covariate categories with small cell sizes (e.g., education, marital status, caregiver type, and comorbidity burden) and excluding an extremely imbalanced variable (dialysis frequency) to assess the robustness of MTM-related estimates. Statistical significance was defined as *p <* 0.05.

### 2.5 Ethical considerations

The study protocol was approved by the Ethics Committee of the First Affiliated Hospital of Shandong First Medical University [Approval No.: YXLL-KY-2025(118)] and adhered to the principles outlined in the Declaration of Helsinki throughout the research process. All participants provided written informed consent.

## 3 Results

### 3.1 Participant characteristics

A total of 274 valid questionnaires were collected. Participant sociodemographic and dialysis-related characteristics are summarized in Table 1. The sample included 153 (55.8%) males and 121 (44.2%) females, with a mean age of 49.18 ± 11.36 years. Most participants were married (85.0%, *n* = 233). Regarding dialysis frequency, 268 (97.8%) patients received dialysis three times per week, while 6 (2.2%) received dialysis five times every 2 weeks.

**Table 1:** Demographic characteristics of participants (*N* = 274).

Variables	Mean ± SD	*n* (%)
Age (years)	49.18 ± 11.36	
Dialysis vintage	4.73 ± 3.55	
Gender		
Male		153 (55.8)
Female		121 (44.2)
Dialysis frequency		
3 times/week		268 (97.8)
5 times/2 weeks		6 (2.2)
Educational level		
Elementary school or below		21 (7.7)
Junior High School		70 (25.5)
High school/Vocational High School		89 (32.5)
University/Junior college		90 (32.8)
Postgraduate (master’s or above)		4 (1.5)
Marital status		
Married		233 (85.0)
Divorced		9 (3.3)
Single		25 (9.1)
Widowed		7 (2.6)
Employment status		
Employed		102 (37.2)
Retirement		51 (18.6)
Freelance occupation		38 (13.9)
Unemployed		83 (30.3)
Primary caregiver		
Spouse		225 (82.1)
Adult children		8 (2.9)
Parents		19 (6.9)
Living alone		22 (8.0)
Monthly income (RMB)		
<1,000		98 (35.8)
1,000–3,000		20 (7.3)
3,000–5,000		59 (21.5)
>5,000		97 (35.4)
Number of comorbidities		
0		127 (46.4)
1		102 (37.2)
2		28 (10.2)
3		10 (3.6)
4		7 (2.6)

### 3.2 Descriptive statistics and reliability of MTM constructs

Table 2 presents descriptive statistics and internal consistency coefficients for all constructs of the MTM. Within the initiation constructs, the mean score for dialogue advantages was 12.40 ± 3.39, and for dialogue disadvantages, it was 5.10 ± 2.56. Behavioral confidence averaged 7.68 ± 4.94, and changes in physical environment averaged 7.00 ± 1.76. The mean initiation intention score was 1.78 ± 1.20. The entire initiation subscale demonstrated good internal consistency, with a Cronbach’s alpha of 0.826.

**Table 2:** Descriptive statistics and reliability tests for MTM constructs.

Constructs	Possible range	Observed range	Mean ± SD	Cronbach’s alpha
Initiation	0–4	0–4	1.78 ± 1.20	–
Dialogue advantages	0–20	5–20	12.40 ± 3.39	0.819
Dialogue disadvantages	0–20	0–12	5.10 ± 2.56	0.752
Behavioral confidence	0–20	0–18	7.68 ± 4.94	0.946
Changes in physical environment	0–12	0–11	7.00 ± 1.76	0.726
Entire initiation scale	–	–	–	0.826
Maintenance	0–4	0–4	1.57 ± 1.13	–
Emotional transformation	0–12	0–12	6.49 ± 2.56	0.854
Practice for change	0–12	0–11	4.90 ± 2.12	0.805
Changes in social environment	0–12	0–12	7.69 ± 2.11	0.749
Entire maintenance scale	–	–	–	0.896
Entire scale	–	–	–	0.908

For the maintenance constructs, mean scores were as follows: emotional transformation 6.49 ± 2.56, practice for change 4.90 ± 2.12, and changes in social environment 7.69 ± 2.11. The mean maintenance intention score was 1.57 ± 1.13. The maintenance subscale showed strong internal consistency (Cronbach’s alpha = 0.896). The total scale exhibited excellent reliability, with a Cronbach’s alpha of 0.908.

### 3.3 Structural validity of the MCPAQ in MHD patients

**Figure 1:**
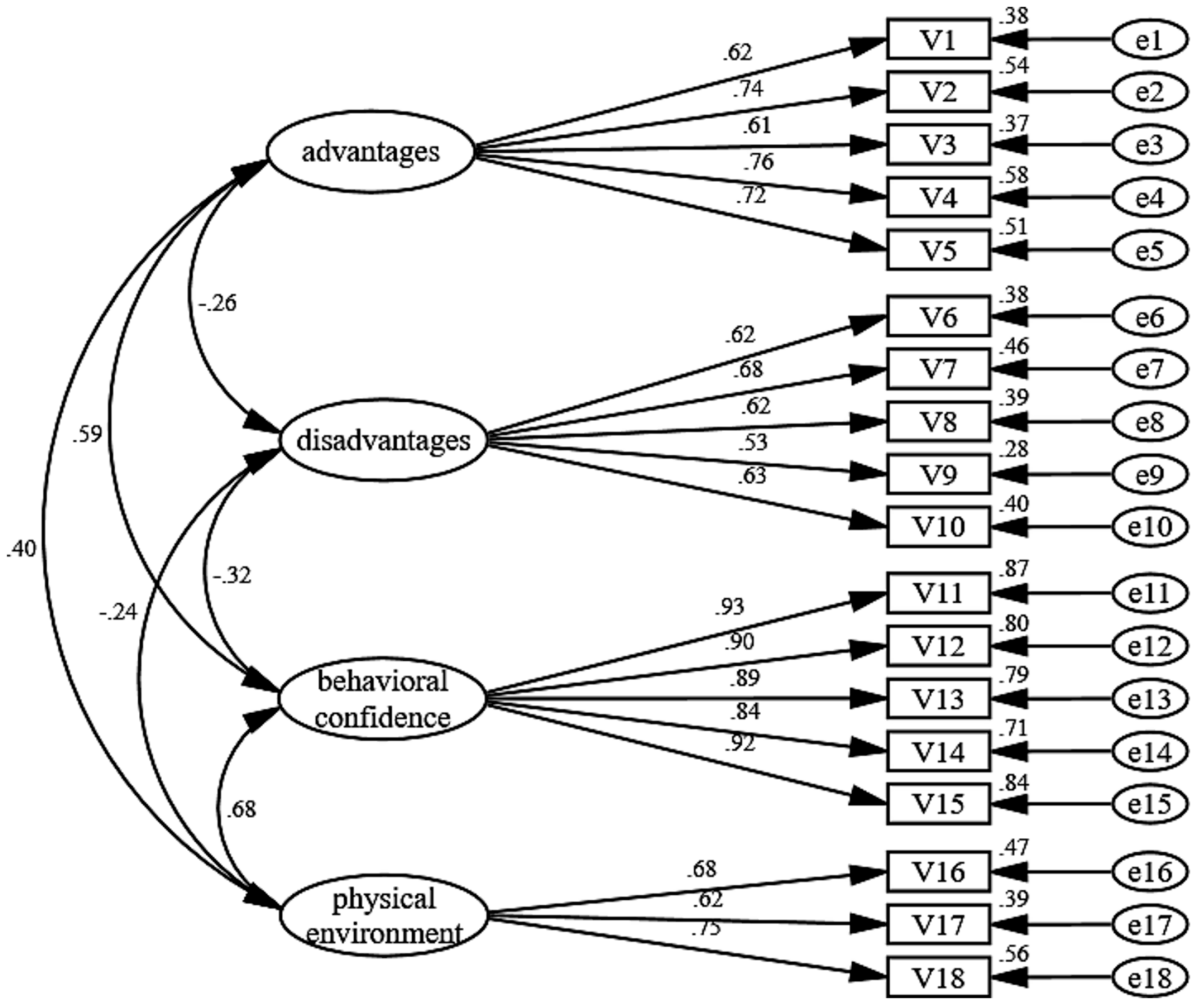
Structural equation model for initiation model.

**Figure 2:**
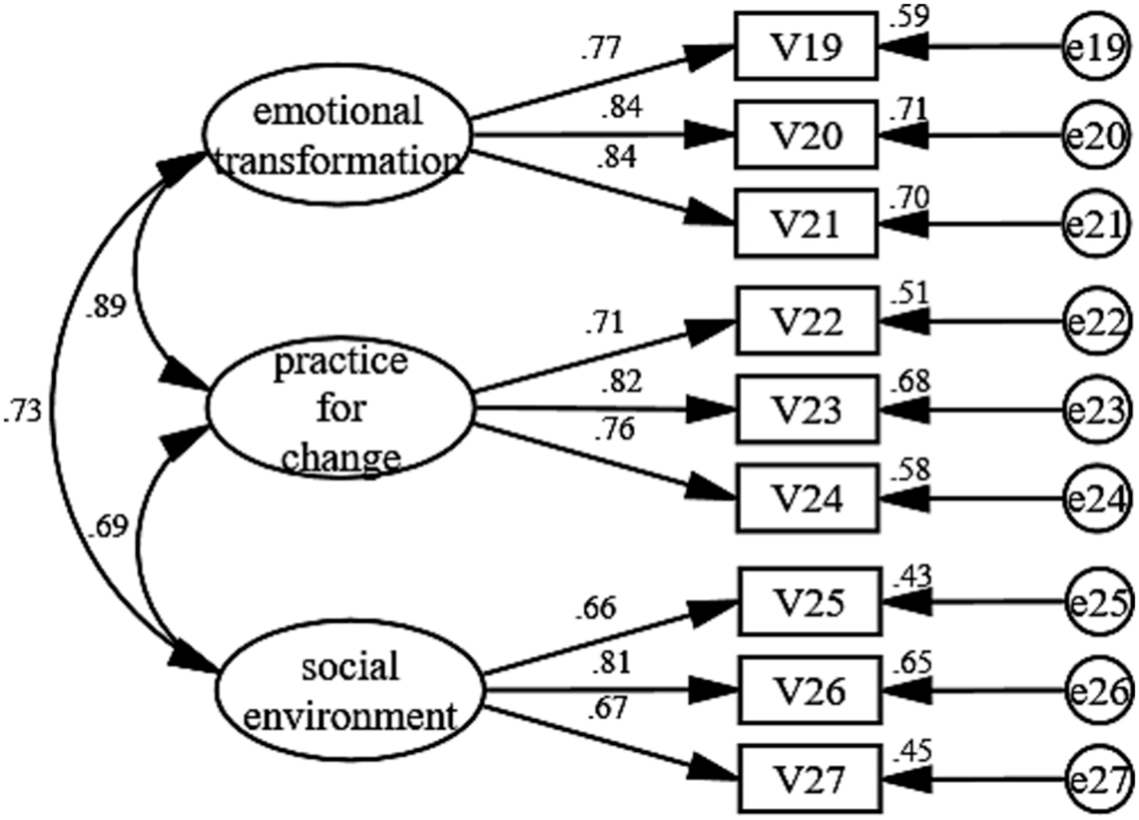
Structural equation model for maintenance model.

To evaluate the structural validity of the MCPAQ in MHD patients, confirmatory factor analysis (CFA) was conducted for the initiation and maintenance models. The standardized CFA measurement models are illustrated in Figures 1, 2.

#### 3.3.1 Model fit

Overall model fit indices are reported in Table 3. For the initiation model, fit indices indicated acceptable model fit (*χ*^2^/df = 2.114, RMSEA = 0.064, CFI = 0.945, NFI = 0.901, IFI = 0.945, TLI = 0.935). Although GFI was slightly below the 0.90 benchmark (GFI = 0.898), the overall fit was considered acceptable given the performance of the primary fit indices. For the maintenance model, fit indices demonstrated excellent model fit (*χ*^2^/df = 1.575, RMSEA = 0.046, GFI = 0.971, CFI = 0.988, NFI = 0.969, IFI = 0.989, TLI = 0.983), supporting the three-factor structure in the MHD sample.

**Table 3:** CFA model fit and convergent validity (initiation and maintenance model).

Phase	Construct	Items (*n*)	Std. loading range	CR	AVE	Model fit indices
Initiation	Dialogue Advantages	5	0.610–0.763	0.820	0.480	*χ*^2^/df = 2.114; RMSEA = 0.064; GFI = 0.898; CFI = 0.945; NFI = 0.901; IFI = 0.945; TLI = 0.935
	Dialogue Disadvantages	5	0.532–0.681	0.756	0.384	
	Behavioral confidence	5	0.843–0.931	0.953	0.802	
	Physical environment	3	0.621–0.746	0.726	0.470	
Maintenance	Emotional transformation	3	0.768–0.845	0.858	0.668	*χ*^2^/df = 1.575; RMSEA = 0.046; GFI = 0.971; CFI = 0.988; NFI = 0.969; IFI = 0.989; TLI = 0.983
	Practice for change	3	0.711–0.825	0.810	0.588	
	Social environment	3	0.656–0.806	0.756	0.510	

All standardized factor loadings were statistically significant (*p <* 0.001).

#### 3.3.2 Factor loadings and convergent validity

Item-level standardized factor loadings and convergent validity indices are shown in Table 3. Standardized loadings across both models ranged from 0.532 to 0.931, indicating that items adequately represented their intended latent constructs. Convergent validity was further supported by composite reliability (CR), which ranged from 0.726 to 0.953 across dimensions. Average variance extracted (AVE) values were > 0.50 for all maintenance constructs (0.668, 0.588, 0.510) and for Behavioral Confidence in the initiation model (0.802). For Dialogue Advantages (0.480) and Changes in Physical Environment (0.470) in the initiation model, AVE values were close to the conventional criterion, while Dialogue Disadvantages showed a lower AVE (0.384).

#### 3.3.3 Discriminant validity

Discriminant validity results using the Fornell–Larcker criterion are presented in Table 4. The initiation constructs demonstrated satisfactory discriminant validity. In the maintenance model, Emotional Transformation and Practice for Change were highly correlated (r = 0.892), exceeding the square roots of their AVE values (0.817 and 0.767, respectively), indicating that discriminant validity between these two constructs did not meet the Fornell–Larcker criterion in this sample.

**Table 4:** Discriminant validity (initiation and maintenance model).

(A) Initiation model				
Constructs	Physical environment	Dialogue advantages	Dialogue disadvantages	Behavioral confidence
Physical environment	0.686	–	–	–
Dialogue advantages	0.405***	0.693	–	–
Dialogue disadvantages	−0.238**ᵇ	−0.264**ᵃ	0.620	–
Behavioral confidence	0.677***	0.594***	−0.324***	0.896

(B) Maintenance model				
Constructs	Social environment	Practice for change	Emotional transformation	
Social environment	0.714	—	—	
Practice for change	0.686***	0.767	—	
Emotional transformation	0.729***	0.892***	0.817	

Diagonal values represent the square root of AVE. Off-diagonal values report standardized latent construct correlations obtained from the CFA measurement model (AMOS). Significance levels: *p < 0.05, **p < 0.01, ***p < 0.001. Exact *p*-values for marked correlations: ᵃ*p* = 0.003; ᵇ*p* = 0.006.

### 3.4 Correlation analysis of MTM constructs

Pearson’s correlation coefficients among MTM constructs are reported in Table 5. Within initiation constructs, behavioral confidence was moderately positively correlated with dialogue advantages (r = 0.527, *p <* 0.01) and changes in physical environment (r = 0.554, *p <* 0.01). Dialogue disadvantages were negatively correlated with other variables. Among initiation variables, initiation intention showed the strongest positive correlation with behavioral confidence (r = 0.838, *p <* 0.01), followed by dialogue advantages (r = 0.626, *p <* 0.01), changes in physical environment (r = 0.590, *p <* 0.01) and dialogue disadvantages (r = −0.332, *p <* 0.01).

**Table 5:** Pearson correlations among constructs in the MTM.

No.	Constructs	1	2	3	4	5
I-1	Dialogue advantages	1				
I-2	Dialogue disadvantages	−0.212**	1			
I-3	Behavioral confidence	0.527**	−0.280**	1		
I-4	Changes in physical environment	0.309**	−0.175**	0.554**	1	
I-5	Initiation	0.626**	−0.332**	0.838**	0.590**	1
M-1	Emotional transformation	1				
M-2	Practice for change	0.754**	1			
M-3	Changes in social environment	0.594**	0.549**	1		
M-4	Maintenance	0.791**	0.785**	0.608**	1	-

Pearson correlations are shown (**p < 0.01). The correlation between initiation intention and maintenance intention (not displayed in the matrix) is r = 0.889 (*p <* 0.01).

Within maintenance constructs, practice for change was strongly correlated with emotional transformation (r = 0.754, *p <* 0.01). Changes in social environment showed moderate positive correlations with emotional transformation (r = 0.594, *p <* 0.01) and practice for change (r = 0.549, *p <* 0.01). Maintenance intention was most strongly correlated with emotional transformation (r = 0.791, *p <* 0.01), followed by practice for change (r = 0.785, *p <* 0.01) and changes in social environment (r = 0.608, *p <* 0.01). Additionally, initiation intention and maintenance intention were strongly positively correlated (r = 0.889, *p <* 0.01).

### 3.5 Hierarchical multiple regression analysis: predictors of initiation and maintenance of PA behavior

Tables 6, 7 presents the results of hierarchical multiple regression analyses examining the predictive effects of MTM constructs on the initiation and maintenance of PA behavior in MHD patients.

**Table 6:** Results of hierarchical multiple regression models predicting initiation intention of PA in MHD patients.

Variables	Model 1					Model 2				
	B	Beta	95% CI	*P*	VIF	B	Beta	95% CI	*P*	VIF
Constant	1.233		[−0.76, 3.23]	0.225		−0.801		[−1.92, 0.32]	0.160	
Gender: Male (Ref: Female)	0.194	0.080	[−0.09, 0.48]	0.176	1.148	0.074	0.031	[−0.07, 0.22]	0.319	1.156
Dialysis frequency: 3 times/week (Ref: 5 times/2 weeks)	0.453	0.055	[−0.48, 1.39]	0.340	1.095	0.236	0.029	[−0.25, 0.72]	0.336	1.102
Age (years)	−0.008	−0.078	[−0.03, 0.01]	0.441	3.314	−0.009	−0.087	[−0.02, 0.00]	0.095	3.323
Educational level (Ref: Elementary school or below)										
Junior High School	0.226	0.082	[−0.36, 0.81]	0.444	3.776	0.246	0.089	[−0.06, 0.55]	0.111	3.859
High school/Vocational High School	0.094	0.037	[−0.51, 0.70]	0.760	4.740	0.017	0.007	[−0.30, 0.33]	0.915	4.788
University/Junior college	−0.047	−0.018	[−0.68, 0.59]	0.884	5.221	0.008	0.003	[−0.32, 0.34]	0.963	5.252
Postgraduate (master’s or above)	0.453	0.045	[−0.83, 1.73]	0.487	1.385	0.336	0.034	[−0.33, 1.00]	0.321	1.413
Monthly income (Ref: <1,000)										
1,000–3,000	−0.483	−0.105	[−1.14, 0.18]	0.150	1.722	0.190	0.041	[−0.15, 0.53]	0.277	1.761
3,000–5,000	0.138	0.047	[−0.39, 0.67]	0.609	2.807	0.121	0.041	[−0.16, 0.40]	0.389	2.840
≥5,000	0.081	0.032	[−0.42, 0.58]	0.749	3.357	0.117	0.047	[−0.14, 0.38]	0.375	3.410
Marital status (Ref: Married)										
Divorced	0.429	0.064	[−0.77, 1.63]	0.482	2.672	−0.192	−0.029	[−0.82, 0.43]	0.545	2.753
Single	0.362	0.087	[−0.88, 1.60]	0.565	7.467	−0.123	−0.029	[−0.77, 0.52]	0.707	7.610
Widowed	0.702	0.092	[−0.57, 1.98]	0.279	2.372	0.178	0.023	[−0.48, 0.84]	0.595	2.398
Employment status (Ref: Employed)										
Retirement	0.031	0.010	[−0.50, 0.56]	0.907	2.500	−0.031	−0.010	[−0.31, 0.24]	0.823	2.511
Freelance occupation	−0.351	−0.101	[−0.82, 0.12]	0.139	1.524	−0.103	−0.030	[−0.35, 0.14]	0.401	1.549
Unemployed	−0.288	−0.110	[−0.83, 0.26]	0.297	3.647	0.008	0.003	[−0.27, 0.29]	0.955	3.683
Primary caregiver (Ref: Living alone)										
Spouse	0.682	0.218	[−0.43, 1.79]	0.226	10.585	−0.124	−0.040	[−0.7, 0.45]	0.672	10.777
Adult children	0.067	0.009	[−1.03, 1.16]	0.905	1.979	−0.125	−0.017	[−0.69, 0.44]	0.667	2.033
Parents	0.826	0.175	[−0.07, 1.72]	0.070	3.024	−0.174	−0.037	[−0.65, 0.30]	0.469	3.205
Dialysis vintage	0.021	0.063	[−0.02, 0.06]	0.310	1.253	0.018	0.053	[0.00, 0.04]	0.103	1.273
Number of comorbidities	−0.449	−0.355	[−0.6, −0.30]	**<0.001**	1.231	−0.084	−0.066	[−0.17, 0.00]	0.051	1.417
Dialogue advantages						0.084	0.238	[0.06, 0.11]	**<0.001**	1.606
Dialogue disadvantages						−0.041	−0.087	[−0.07, −0.01]	**0.006**	1.223
Behavioral confidence						0.136	0.560	[0.12, 0.16]	**<0.001**	2.250
Changes in physical environment						0.122	0.180	[0.07, 0.17]	**<0.001**	1.753
R^2^	0.230					0.800				
Adjusted R^2^	0.166					0.779				
F	3.591					39.576				
∆R^2^	0.230					0.569				
∆F	3.591					176.104				

Bold values indicate statistical significance at *p* < 0.05.

**Table 7:** Results of hierarchical multiple regression models predicting maintenance intention of PA in MHD patients.

Variables	Model 1					Model 2				
	B	Beta	95% CI	*P*	VIF	B	Beta	95% CI	*P*	VIF
Constant	0.938		[−0.93, 2.81]	0.324		−1.988		[−3.07, −0.91]	**<0.001**	
Gender: Male (Ref: Female)	0.173	0.076	[−0.09, 0.44]	0.199	1.148	0.131	0.058	[−0.02, 0.28]	0.079	1.152
Dialysis frequency: 3 times/week (Ref: 5 times/2 weeks)	0.188	0.024	[−0.69, 1.06]	0.673	1.095	0.380	0.049	[−0.1, 0.86]	0.123	1.099
Age (years)	−0.004	−0.036	[−0.02, 0.02]	0.722	3.314	0.003	0.030	[−0.01, 0.01]	0.589	3.337
Educational level (Ref: Elementary school or below)										
Junior High School	−0.042	−0.016	[−0.59, 0.5]	0.879	3.776	−0.070	−0.027	[−0.37, 0.23]	0.647	3.822
High school/Vocational High School	0.134	0.056	[−0.44, 0.7]	0.644	4.740	0.043	0.018	[−0.27, 0.36]	0.789	4.812
University/Junior college	−0.039	−0.016	[−0.64, 0.56]	0.897	5.221	0.030	0.013	[−0.3, 0.36]	0.856	5.252
Postgraduate (master’s or above)	0.344	0.037	[−0.86, 1.55]	0.573	1.385	0.124	0.013	[−0.54, 0.79]	0.714	1.392
Monthly income (Ref: <1,000)										
1,000–3,000	−0.392	−0.091	[−1.01, 0.23]	0.212	1.722	−0.072	−0.017	[−0.41, 0.27]	0.678	1.736
3,000–5,000	0.139	0.051	[−0.36, 0.64]	0.583	2.807	0.049	0.018	[−0.23, 0.33]	0.728	2.840
≥5,000	−0.097	−0.041	[−0.57, 0.37]	0.684	3.357	−0.174	−0.074	[−0.43, 0.09]	0.189	3.373
Marital status (Ref: Married)										
Divorced	0.491	0.078	[−0.63, 1.61]	0.389	2.672	0.172	0.027	[−0.46, 0.8]	0.593	2.774
Single	0.027	0.007	[−1.14, 1.19]	0.964	7.467	0.544	0.139	[−0.1, 1.19]	0.098	7.569
Widowed	0.297	0.042	[−0.9, 1.49]	0.625	2.372	−0.069	−0.010	[−0.73, 0.59]	0.838	2.383
Employment status (Ref: Employed)										
Retirement	−0.083	−0.029	[−0.58, 0.42]	0.744	2.500	−0.116	−0.040	[−0.39, 0.16]	0.411	2.539
Freelance occupation	−0.068	−0.021	[−0.51, 0.37]	0.760	1.524	0.168	0.052	[−0.08, 0.41]	0.177	1.562
Unemployed	−0.280	−0.114	[−0.79, 0.23]	0.280	3.647	−0.062	−0.025	[−0.34, 0.22]	0.667	3.678
Primary caregiver (Ref: Living alone)										
Spouse	0.825	0.281	[−0.21, 1.87]	0.119	10.585	0.354	0.121	[−0.23, 0.94]	0.235	11.032
Adult children	0.174	0.026	[−0.85, 1.2]	0.737	1.979	0.313	0.047	[−0.26, 0.89]	0.286	2.068
Parents	1.449	0.327	[0.61, 2.29]	**0.001**	3.024	−0.149	−0.034	[−0.65, 0.35]	0.554	3.495
Dialysis vintage	0.031	0.099	[−0.01, 0.07]	0.111	1.253	0.028	0.087	[0.01, 0.05]	**0.012**	1.267
Number of comorbidities	−0.394	−0.332	[−0.54, −0.25]	**<0.001**	1.231	−0.165	−0.139	[−0.25, −0.08]	**<0.001**	1.361
Emotional transformation						0.172	0.390	[0.13, 0.22]	**<0.001**	3.027
Practice for change						0.212	0.398	[0.16, 0.26]	**<0.001**	2.632
Changes in social environment						0.070	0.131	[0.02, 0.12]	**0.004**	2.237
R^2^	0.231					0.769				
Adjusted R^2^	0.167					0.746				
F	3.612					34.477				
∆R^2^	0.231					0.537				
∆F	3.612					192.793				

Bold values indicate statistical significance at *p* < 0.05.

#### 3.5.1 Predictors of PA initiation intention

Model 1, which included only covariates, explained 16.6% of the variance in initiation intention (adjusted R^2^ = 0.166, *F* = 3.591, *p <* 0.001). Among covariates, the number of comorbidities was negatively associated with initiation intention (*β* = −0.355, *p <* 0.001), indicating that greater comorbidity burden was linked to lower PA initiation intention. After adding MTM initiation constructs (dialogue advantages, dialogue disadvantages, behavioral confidence, and changes in physical environment) in Model 2, the explained variance increased significantly by 56.9% (ΔR^2^ = 0.569, ΔF = 176.104, *p <* 0.001). Behavioral confidence was the strongest predictor (*β* = 0.560, *p <* 0.001), followed by dialogue advantages (*β* = 0.238, *p <* 0.001), changes in physical environment (*β* = 0.180, *p <* 0.001) and dialogue disadvantages (*β* = −0.087, *p* = 0.006). The number of comorbidities was no longer significant after adjusting for other variables.

#### 3.5.2 Predictors of PA maintenance intention

In Model 1, examining maintenance intention with only covariates, 16.7% of variance was explained (adjusted R^2^ = 0.167, *F* = 3.612, *p <* 0.001). Number of comorbidities negatively predicted maintenance intention (*β* = −0.332, *p <* 0.001), whereas having a primary caregiver who was a parent showed a positive association (*β* = 0.327, *p* = 0.001). Model 2, incorporating MTM maintenance constructs (emotional transformation, practice for change, and changes in social environment), improved the explained variance by 53.7% (ΔR^2^ = 0.537, ΔF = 192.793, *p <* 0.001). Emotional transformation (*β* = 0.390, *p <* 0.001) and practice for change (*β* = 0.398, *p <* 0.001) were the most salient positive predictors, while changes in social environment also contributed significantly but to a lesser degree (*β* = 0.131, *p* = 0.004). In the final model, the number of comorbidities (*β* = −0.139, *p <* 0.001) and dialysis vintage (*β* = 0.087, *p* = 0.012) were significant, but the effect of caregiver type was no longer significant. Some covariate categories were infrequent, which may yield imprecise regression estimates for those subgroups. Sensitivity analyses using collapsed covariate categories yielded materially unchanged MTM construct estimates for both initiation and maintenance intentions (Supplementary Table S1,A,B).

## 4 Discussion

This study applies the MTM to examine factors related to PA behavior change among MHD patients. Our findings demonstrate that the six core constructs of the MTM significantly predict both the initiation and sustenance intentions of PA in this population.

The MCPAQ demonstrated overall acceptable measurement performance in this MHD sample, providing preliminary support for its use in interpreting MTM-based findings. Importantly, two construct-level patterns warrant contextual interpretation. First, the relatively low AVE for the Dialogue Disadvantages factor may reflect the multifaceted nature of PA barriers in MHD patients (e.g., physical limitations, fatigue concerns, time conflicts, and psychological worries). Such heterogeneous barriers may not be fully captured by the existing items, resulting in lower shared variance even when the construct remains clinically meaningful. Second, the relatively high association between Emotional Transformation and Practice for Change suggests that, during PA maintenance in MHD patients, affective reinforcement from PA and concrete self-regulation practices may function as closely intertwined processes. Thus, although the overall maintenance measurement model was supported, these two subdimensions should be interpreted cautiously as partially overlapping components in this clinical context. Future studies should refine items and re-examine discriminant validity in larger, independent samples.

### 4.1 Predictive effects of MTM constructs on PA initiation intention

In the present study, dialogue advantages and dialogue disadvantages were examined as distinct constructs rather than as a composite difference score, allowing a more nuanced interpretation of how outcome appraisals relate to initiation intention. Both dimensions were associated with initiation intention. Overall, initiation intention may be more closely aligned with perceived benefits, such as anticipated improvements in physical functioning and quality of life (Jayaseelan et al., 2018), while perceived disadvantages remain relevant as distinct concerns, such as discomfort during activity (Zhang et al., 2025) or worries about potential impacts on arteriovenous fistulas (Li et al., 2021; Lightfoot et al., 2021). Collectively, these findings support treating perceived advantages and perceived disadvantages as coexisting cognitive pathways rather than a single continuum.

Behavioral confidence was strongly associated with initiation intention, suggesting that confidence-related processes may be salient for intention formation among MHD patients. Previous studies indicate that MHD patients often experience physiological constraints, including fatigue (Chang et al., 2025), muscle wasting (Wang et al., 2023), and impaired cardiopulmonary function (Pella et al., 2024). Such constraints may be linked to reduced confidence in performing moderate-intensity PA and may relate to lower initiation intention. In our sample, behavioral confidence levels appeared relatively low. Strategies such as personalized progressive activity plans (Battaglia et al., 2024) may help patients gradually experience success and boost their confidence. Taken together, these findings suggest that confidence-related processes warrant further investigation in longitudinal and experimental studies to evaluate their potential relevance for intervention development.

Changes in the physical environment were also associated with initiation intention, suggesting that intention formation in MHD patients may relate not only to psychological factors but also to external environmental support, consistent with prior studies (Li et al., 2021). Given the time constraints of dialysis sessions and the unique physical conditions of MHD patients, access to convenient facilities, appropriate equipment, and safe environments may be relevant to PA-related intention formation in this population (Li et al., 2021; Bennett et al., 2022). Future research could explore whether enhancing access to safe and feasible low-intensity activity options, including those embedded in dialysis-related settings, is associated with stronger initiation intentions and subsequent PA participation.

### 4.2 Predictive effects of MTM constructs on PA maintenance intention

For maintenance intention, emotional transformation was associated with the intention to sustain guideline-level PA over time, suggesting that affect-related processes may be relevant to maintenance-stage intention. This interpretation is consistent with prior work (Huang et al., 2023) that engagement in PA can be accompanied by perceived improvements in symptoms, functioning, and mood, which may provide affective reinforcement for continued participation. Future intervention studies could test whether approaches that enhance recognition of positive affective experiences during PA are associated with stronger maintenance intentions. In MHD patients, affect-related reinforcement may co-occur with self-regulation efforts; therefore, emotional transformation and practice for change may represent closely related (but conceptually distinct) components of a broader maintenance process, warranting further validation to strengthen construct distinctiveness in this clinical context.

Practice for change was also associated with maintenance intention. This pattern suggests that patients’ capacity to adapt activity strategies to individual conditions and manage barriers may be relevant to maintenance-stage intention. Multifaceted barriers have been reported to interfere with longer-term PA engagement in hemodialysis populations. The existing literature indicates that physiological burdens, such as post-dialysis fatigue, psychosocial hurdles (e.g., insufficient motivation), and environmental constraints (e.g., time availability and weather conditions) hinder long-term engagement (Battaglia et al., 2024). Future intervention studies may evaluate whether structured skill-building components, such as goal setting, graded tasks, feedback, and problem solving, are associated with improvements in maintenance intention and longer-term PA engagement (Carraça et al., 2021).

Changes in social environment were associated with maintenance intention, consistent with evidence that social support from family members, friends, and healthcare professionals can relate to PA-related motivation in MHD patients (Zhang et al., 2025). At the same time, social influences may not be uniformly facilitative; family beliefs and norms may shape whether activity is encouraged or constrained in some contexts (Huang et al., 2023). This phenomenon suggests that addressing familial perceptions might be a relevant aspect when considering social support for PA intention. Future studies could examine how different sources and forms of support (e.g., peer support groups, family-focused education, and follow-up support from healthcare teams) relate to intentions and behavior over time (Longley et al., 2023; Anita et al., 2025; Suminski et al., 2024).

To avoid ambiguity, we clarify that “maintenance intention” in this study refers to a forward-looking intention to sustain guideline-level PA (≥150 min/week) going forward, rather than maintenance of baseline PA levels. Notably, a significant correlation was found between initiation and maintenance intentions. Accordingly, future intervention studies could explore whether incorporating maintenance-supportive elements during the initiation phase is associated with stronger intentions across stages.

### 4.3 Impact of demographic and clinical variables

Before incorporating MTM constructs, covariates explained only approximately 17% of the variance in initiation and maintenance intentions, suggesting that demographic and clinical factors have limited predictive capacity for behavioral intentions. Notably, a higher number of comorbidities was significantly associated with lower maintenance intention in the final model. For initiation intention, the significant association observed in the demographic model was attenuated (*p* = 0.051) after adjusting for MTM constructs. This pattern suggests that the relationship between comorbidities and initiation intention may be partially explained by psychological factors, such as behavioral confidence, whereas the physical burden of comorbidities appears to be an independent correlate of maintenance intention. Comorbidities are often linked to increased disease severity (Wu et al., 2022a) and psychological burden (Wu et al., 2022b), both of which may influence PA intentions. The association between dialysis vintage and maintenance intention may be explained by the observation that patients undergoing short-term dialysis perceive more severe dialysis-related symptoms and demonstrate poorer adaptation to the disease and treatment compared to their long-term counterparts (Chang and Kim, 2025), potentially influencing the intention to sustain PA. Other demographic variables did not exhibit significant predictive effects after the inclusion of MTM constructs.

However, approximately 23–25% of unexplained variance suggests that additional unmeasured factors remain critical. Mood disturbances, particularly depression, represent a major potential correlate; evidence indicates a strong inverse correlation between depressive symptoms and self-reported physical activity levels, suggesting that psychological distress may suppress the translation of intent into action (Musolino et al., 2023). Furthermore, fatigue remains a pervasive barrier. Between 60 and 97% of patients on hemodialysis suffer from profound exhaustion, often described as a “vicious cycle” of post-dialysis depletion that severely constrains life participation and motivation (Jacobson et al., 2019). Clinical markers like dialysis adequacy also play a role, although the relationship is complex; surprisingly, higher equilibrated Kt/V has been associated with lower physical activity levels, possibly reflecting the interplay between intensive clearance, muscle mass, and physical conditioning (Han et al., 2025). These unmeasured biological and psychological burdens likely constitute the missing links in predicting behavioral intentions in this population.

### 4.4 Theoretical contributions and clinical applications

The theoretical contribution of this study lies in examining the applicability and predictive utility of the MTM for PA intentions among MHD patients. MTM conceptualizes behavior change as a two-stage process (initiation and maintenance) and posits that determinants may differ by stage. Our findings provide preliminary evidence consistent with this proposition, suggesting that initiation and maintenance related intentions may be associated with different sets of factors.

In terms of clinical implications, this study provides preliminary evidence regarding correlates of PA intention in MHD patients. Within the MTM framework, initiation intention was more closely related to confidence-related, environmental support, and perceived advantages/disadvantages domains, whereas maintenance intention was more closely related to affect-related processes, behavior change skills, and social support. These proposed stage-specific targets require confirmation in longitudinal and intervention studies before being translated into intervention components.

### 4.5 Strengths

This study has several strengths. First, from a theoretical perspective, we applied the MTM to examine PA intention and operationalized its two-stage framework by modeling initiation and maintenance intentions separately, allowing an explicit evaluation of whether associated factors differ by stage. Second, in terms of design and analytic strategy, we examined the incremental contribution of MTM constructs after adjusting for relevant sociodemographic and clinical covariates, facilitating interpretation of the observed associations within the proposed framework. Third, regarding measurement rigor, we evaluated the measurement model using confirmatory factor analysis and assessed measurement quality (model fit, convergent/discriminant validity indicators, and reliability). The initiation model showed acceptable fit with one index slightly below the conventional benchmark (GFI = 0.898), whereas the maintenance model showed good overall fit. While some validity indices did not fully meet conventional benchmarks, this transparent measurement evaluation provides an empirical basis for interpreting the findings appropriately and for refining closely related constructs in future validation work. The subscales demonstrated good internal consistency (Cronbach’s alpha = 0.826 for initiation; 0.896 for maintenance; 0.908 for the overall scale), supporting score reliability.

### 4.6 Limitations

This study has the following limitations. First, due to the cross-sectional design of this study, we cannot infer causality or rule out the possibility of reverse causality between the theoretical constructs and PA intention. While our models hypothesize that factors such as behavioral confidence and supportive social environments contribute to the formation of PA intention, these relationships are likely reciprocal. For instance, a longitudinal study demonstrated that stronger PA intentions at baseline significantly predicted higher perceived social support during follow-up (Paech et al., 2016). Therefore, longitudinal studies are needed to clarify the temporal sequence and causal directions of these associations. Second, the assessment metric was behavioral intention rather than objectively measured PA levels. While intention is theoretically posited as the proximal determinant of action, the well-documented “intention-behavior gap” in health psychology suggests that intention alone often accounts for only a modest proportion of variance in behavioral enactment. For instance, a meta-analysis indicated that nearly half of PA intenders fail to translate their intentions into action (Feil et al., 2023). This discrepancy may be moderated by factors such as executive function, self-efficacy, and planning (Zhu et al., 2022). In the context of MHD, disease-related barriers, such as post-dialysis fatigue or unpredictable fluctuations in health status, may further widen this gap, preventing even highly motivated patients from engaging in PA. Future research should not only use objective PA measures (e.g., accelerometers) but also investigate how post-intentional strategies, such as action control or coping planning, facilitate its bridging for MHD patients (Yang et al., 2025). Third, the convenience sampling from a single tertiary hospital limits the generalizability of the findings. Epidemiological data indicate that in China, approximately 48.7, 38.4, and 12.9% of MHD patients receive treatment in tertiary, secondary, and primary hospitals, respectively (Zhang et al., 2025). Within the tiered healthcare system, tertiary centers typically serve patients with greater clinical complexity. Given our finding that a higher number of comorbidities is associated with lower PA intentions (especially for maintenance), the average PA intention in our sample is likely to be slightly lower than that of the broader MHD population. Nevertheless, the significant predictive relationships identified within this sample underscore the robustness of the theoretical model in this key patient subset. Importantly, this potential sampling bias may have influenced the magnitude of associations. Future research should employ stratified sampling across different levels of healthcare facilities to verify generalizability and estimate more precise effect sizes. Fourth, although the model explained a substantial proportion of variance, approximately 23–25% remained unexplained, suggesting the influence of other psychological, sociocultural, or clinical factors warranting further investigation. Our sample was restricted to patients using a native AVF, which may limit generalizability to individuals using other vascular access types. In addition, several potentially relevant variables were not comprehensively assessed, including specific comorbidity types, pre-CKD PA history, fatigue severity, and dialysis-related parameters. Future studies should incorporate more detailed clinical phenotyping and historical PA information, test whether these factors moderate or mediate intention formation, and further improve the explanatory power of the model. In addition, future studies should replicate the CFA in independent MHD samples and further evaluate the distinctiveness of closely related maintenance constructs to strengthen measurement precision.

## 5 Conclusion

This study demonstrates the utility of the MTM in predicting PA intentions among MHD patients, particularly in identifying factors associated with the initiation and maintenance stages. The MTM framework effectively captures the complexity of behavior change, providing a solid theoretical foundation for future intervention strategies. Although cross-sectional in nature and unable to infer causality, the identified key factors offer valuable insights for intervention design. Future research should employ longitudinal designs and objective PA measurements to further validate these findings.

## Data Availability

The raw data supporting the conclusions of this article will be made available by the authors, without undue reservation.
