# Supplementary material for: Predictive utility of the multi-theory model in physical activity initiation and maintenance intentions among maintenance hemodialysis patients: a cross-sectional study
Source: Front Psychol. 2026 Mar 6;17:1752207. doi: 10.3389/fpsyg.2026.1752207 (PMC13002428; doi:10.3389/fpsyg.2026.1752207)
Supplement: Supplementary file 1 [file Table_1.docx]

**Supplementary Table S1. Sensitivity analysis of hierarchical regression models predicting PA initiation and maintenance intentions among MHD patients**

**Panel A. Initiation intention**

| **MTM construct** | **Primary analysis (Model 2) Std Beta** | **Primary analysis (Model 2) P** | **Sensitivity analysis (Model 2) Std Beta** | **Sensitivity analysis (Model 2) P** |
| --- | --- | --- | --- | --- |
| Dialogue advantages | 0.238 | <0.001 | 0.242 | <0.001 |
| Dialogue disadvantages | -0.087 | 0.006 | -0.095 | 0.002 |
| Behavioral confidence | 0.560 | <0.001 | 0.563 | <0.001 |
| Changes in physical environment | 0.180 | <0.001 | 0.164 | <0.001 |

| **Model summary (Model 2)** | **Primary** | **Sensitivity** |
| --- | --- | --- |
| R² | 0.800 | 0.791 |
| Adjusted R² | 0.779 | 0.777 |
| ΔR² (Model 2 vs Model 1) | 0.569 | 0.602 |

**Panel B. Maintenance intention**

| **MTM construct** | **Primary analysis (Model 2) Std Beta** | **Primary analysis (Model 2) P** | **Sensitivity analysis (Model 2) Std Beta** | **Sensitivity analysis (Model 2) P** |
| --- | --- | --- | --- | --- |
| Emotional transformation | 0.390 | <0.001 | 0.379 | <0.001 |
| Practice for change | 0.398 | <0.001 | 0.400 | <0.001 |
| Changes in social environment | 0.131 | 0.004 | 0.138 | 0.002 |

| **Model summary (Model 2)** | **Primary** | **Sensitivity** |
| --- | --- | --- |
| R² | 0.769 | 0.759 |
| Adjusted R² | 0.746 | 0.744 |
| ΔR² (Model 2 vs Model 1) | 0.537 | 0.590 |

**Footnotes:**

1. Values are standardized regression coefficients (Std Beta) from Model 2. P values are reported as provided by the software (e.g., 0.000 is shown as P < 0.001).

2. Primary analysis corresponds to the main hierarchical regression models reported in the manuscript.

3. Sensitivity analysis re-estimated the models after collapsing sparse covariate categories to improve estimate stability; the set of MTM constructs included in Model 2 was unchanged.
